# Litterfall production and fine root dynamics in cool-temperate forests

**DOI:** 10.1371/journal.pone.0180126

**Published:** 2017-06-29

**Authors:** Ji Young An, Byung Bae Park, Jung Hwa Chun, Akira Osawa

**Affiliations:** 1 Division of Forest Ecology, Korea Forest Research Institute, Seoul, Republic of Korea; 2 Division of Forest and Biomaterials Science, Kyoto University, Kyoto, Japan; The University of Auckland, NEW ZEALAND

## Abstract

Current understanding of litterfall and fine root dynamics in temperate forests is limited, even though these are the major contributors to carbon and nutrient cycling in the ecosystems. In this study, we investigated litterfall and fine root biomass and production in five deciduous and four coniferous forests at the Gwangneung Experimental Forest in Korea. We used ingrowth cores to measure fine root production and root turnover rate. The litterfall was separated into leaves, twigs, and others, and then leaves were further separated according to species. Annual litterfall mass was not significantly different between the years, 360 to 651 g m^-2^ in 2011 and 300 to 656 g m^-2^ in 2012. Annual fine root (<5 mm) production was significantly higher in 2012 (421 to 1342 g m^-2^) than in 2011 (99 to 872 g m^-2^). Annual litterfall mass was significantly different among the stands, while fine root production did not statistically differ among the stands. The average fine root turnover rate, calculated by dividing the annual fine root production by the maximum standing fine root biomass, was 1.65 for deciduous forests and 1.97 for coniferous forests. Fine root production constituted 18–44% of NPP, where NPP was the sum of woody biomass production, litterfall production, and fine root production. Belowground production was a greater fraction of NPP in more productive forests suggesting their greater carbon allocation belowground.

## Introduction

Tree roots and aboveground vegetation are huge carbon and nutrient sinks, and they play an important role in the carbon and nutrient cycles of forest ecosystems. Particularly, litterfall and fine root production are major components of NPP [1,2]. However, our knowledge on the belowground sink strength of forest ecosystems is still limited due to relatively poor knowledge on the process of fine root production [2–4]. This may lead to unreliable estimates of carbon budget and uncertainties in forest simulation models [5–7].

Aboveground litterfall is a key process regulating the carbon and nutrient cycling in forests, but the quantity of fallen leaves greatly depends on spatiotemporal factors and is determined by climate, topography, species composition, and soil fertility [8–10]. Zhang et al. [11] conducted a literature study of over 400 reported studies and monographs on seasonal litterfall in various types of forest ecosystems on a global scale, and reported that the seasonal patterns of litterfall in temperate forests are diverse with a peak production in autumn, and that they cannot be characterized by specific environmental variables due to the species diversity and the variability in related environmental conditions.

Belowground processes such as fine root production, mortality, and decomposition, which are less well understood relative to aboveground process, are of great importance because belowground biomass production can be as large as aboveground production [3,4]. Several studies have reported that a high proportion of the annual NPP is contributed by fine roots and that the belowground production contributes up to 70% to NPP in some forests [12–15]. Therefore, increased knowledge of the fine root production across various types of temperate forests may lead to a better understanding of its contribution to the forest NPP and the potential role of forests in mitigating climate warming. Furthermore, understanding the underlying mechanisms related to fine root production is one of the most important research objectives in forest ecology.

Understanding the quantitative relationships between above- and belowground production is also important to understand the carbon cycle of forest ecosystems [16–19]. Several attempts have been made to establish the relationships of fine root production and litterfall mass across gradients of water availability [20–22], soil nutrient availability [20,22–25], altitude [2,26], and temperature [27,28]. However, no consistent relationship has been established between above- and belowground production, due to the small number of studies and difficulty in accurately measuring belowground production [21,29–32]. Therefore, it will be meaningful to further increase the number of attempts to estimate litterfall and fine root production simultaneously, and to improve accuracy in the estimates of fine root production.

The objectives of this study were to estimate litterfall and fine root production in various types of temperate forests in Korea and to identify the relationship between above- and belowground production. We used the ingrowth core method to estimate fine root production and turnover rate. We hypothesized that fine root production increases with aboveground productivity because above- and belowground carbon allocation are strongly related with each other and are limited by the same factors. This study will lead to increased knowledge of the relationship between above- and belowground production across various types of temperate forests and may contribute to a better understanding of their contribution to evaluation of ecosystem NPP.

## Materials and methods

### Ethics statement

The study site (Gwangneung Experimental Forest) is managed by Dr. You-Mi Lee of the Korea National Arboretum. All necessary permits were obtained from her for the field sampling and the study did not involve endangered or protected species.

### Site and stand description

This study was conducted in multiple forest types at the Gwangneung Experimental Forest (GEF), about 30 km northeast of Seoul, in the central cool temperate forest sub zone of Korea. The long-term ecological research site of the Gwangneung experimental forest is a well reserved and protected forest in Korea. Natural deciduous forests near the Soribong peak (533 m above sea level [a.s.l.]) in the GEF have been protected from human disturbances and forest management activities because of the presence of a royal tomb. Therefore, the GEF site has drawn extensive attention from many ecologists in Korea [33,34]. However, study on fine root has not been conducted at the GEF, and the above- and belowground forest production has not been investigated simultaneously in this area.

The main study area was composed of natural deciduous forests and coniferous plantations (127°8′31′′–127°10′14′′E and 37°44′30′′–37°45′21′′N; elevation 55–533 m a.s.l.). To estimate forest biomass and to investigate stand structure, nine study plots (five plots in deciduous forests and four plots in coniferous forests) were established in 2005 (Table 1) with various sizes ranging from 0.02 to 0.09 ha to ensure homogeneity within each plot [35]. Major tree species in the deciduous forests are *Quercus serrata*, *Carpinus laxiflora*, *Carpinus cordata*, and *Acer pictum*. The main tree species in the coniferous plantations are *Abies holophylla* (plots 6 and 7), *Abies koreana* (plot 8), and *Pinus koraiensis* (plot 9) [35–37]. The aspect is easterly and the slope is approximately 10°. The parent rock type is granitic gneiss, the soil type is brown forest soil (Alfisol in the USDA system), and the average soil depth is 52 cm. Soil texture is loam or sandy loam and the pH ranges from 4.2 to 5.2 [33,38].

**Table 1:** Main characteristics of the nine GEF study stands [35].

Forest	Stand	Dominant species	Basal	Tree	Woody	Woody biomass
type			area	density	biomass	production
			(m^2^ ha^-1^)	(trees ha^-1^)	(t ha^-1^)	(t ha^-1^)
Deciduous	1	*Quercus serrata*, *Carpinus laxiflora*, *Carpinus cordata*	34.0	1200	295	9.94
	2	*Quercus serrata*, *Carpinus laxiflora*, *Carpinus cordata*	21.5	833	195	5.28
	3	*Quercus serrata*, *Carpinus cordata*	36.1	1350	340	4.79
	4	*Quercus serrata*, *Quercus mongolica*, *Carpinus laxiflora*	29.7	2800	224	7.63
	5	*Quercus serrata*, *Quercus variabilis*, *Carpinus laxiflora*	26.1	1367	230	6.14
Coniferous	6	*Abies holophylla*	35.5	1267	170	7.34
	7	*Abies holophylla*	18.0	1950	155	9.87
	8	*Abies koreana*	28.2	1150	203	7.03
	9	*Pinus koraiensis*	31.8	1525	154	6.82

Woody biomass and production (stems, branches, and coarse roots) were estimated by allometric models [33,35,39].

### Meteorological conditions

**Fig 1:**
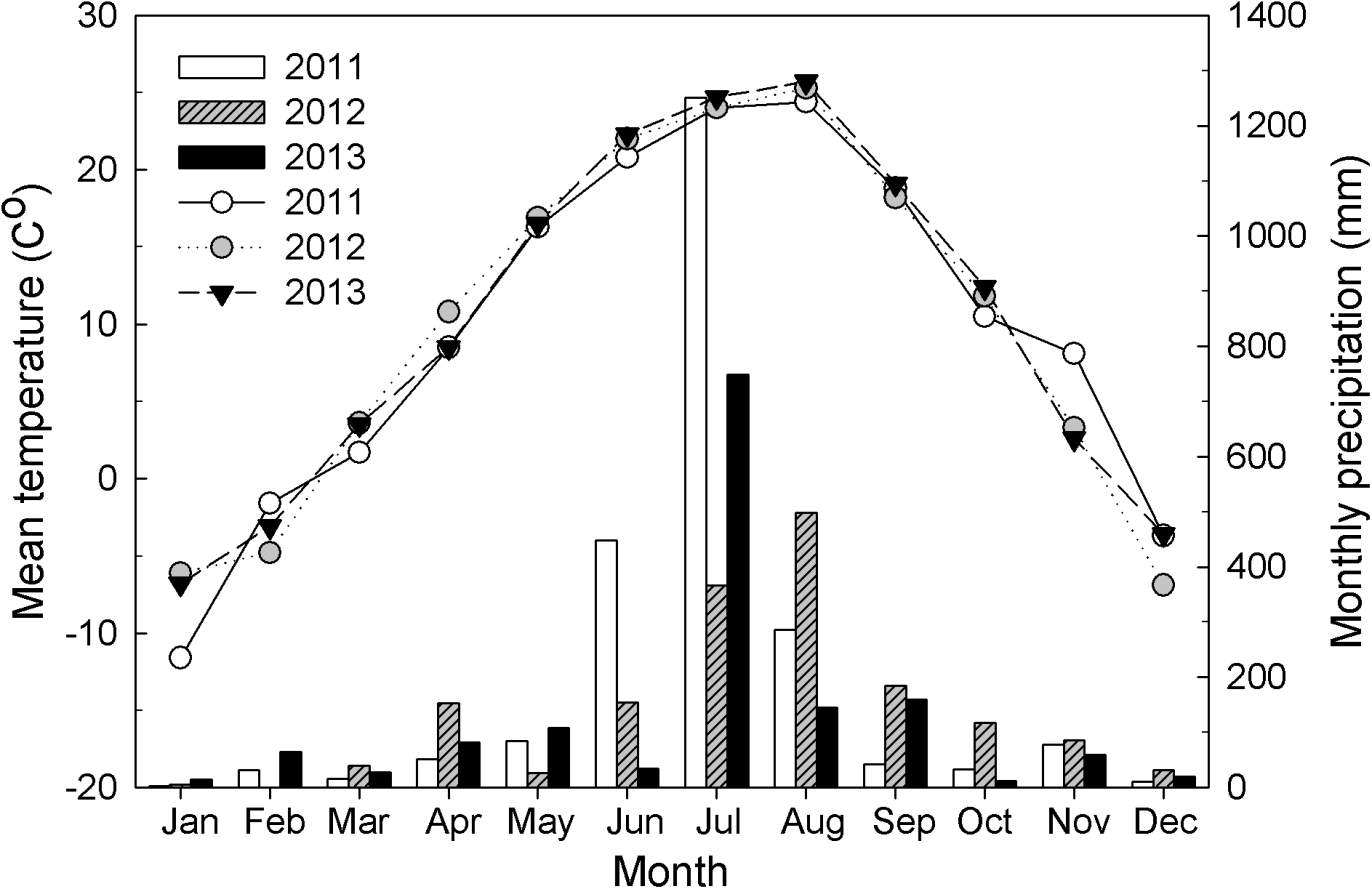
Mean monthly temperature and monthly precipitation in 2011, 2012 and 2013.

Air temperature averaged 11.5°C and precipitation averaged 1,436 mm from 1970 to 2004 [38]. Summers are hot and humid (monsoon climate) and winters are cold and dry (continental climate); approximately 70% of the annual precipitation is concentrated in the period between June and September due to intensive rainfalls in June or July and typhoons from July to September [33,38]. During this study, air temperature and precipitation were measured by the automatic weather station at Gwangneung (37°45′N, 127°10′E; elevation 102 a.s.l.). Mean monthly temperature and monthly precipitation during the study period are shown in Fig 1.

### Soil sampling and analysis

For physical and chemical analyses, soil samples (approximately 500 g) were collected separately from the A (mean depth 7 cm) and B horizons (mean depth 19 cm) down to a maximum depth of 50 cm at two random locations in each plot in July 2011. The sampling points were located at least 1 m from trees and were not disturbed by coarse woody debris and wild animals such as a wild boar. The collected soil samples were stored at 4°C until analysis. To measure the soil bulk density (g m^-3^), A and B soil horizons were sampled separately using a cylindrical core.

Soil analysis was conducted according to National Institute of Agricultural Science and Technology [40] and Sparks et al. [41]. Soil texture was determined by the hydrometer method at 30°C and the soil organic matter (OM) content was determined using the Tyurin method [42,43]. The soil pH and electrical conductivity (EC) were measured using a 1:5 (w/v) soil:distilled water suspension. Total nitrogen (TN) was measured in 1 g soil by using the micro-Kjeldahl method [44] and available phosphorus (P_2_O_5_; AP) was measured with the Lancaster method [45,46]. The cation exchange capacity (CEC) was determined in 1N HN_4_OAc and CH_3_COOH extracts by using the Brown method [47]. Exchangeable cations K^+^, Ca^2+^, Mg^2+^, and Na^+^ in the 1N NH_4_OAc extract were determined using an atomic absorption spectrometer (AA280FS; Agilent Technologies, Santa Clara, CA, U.S.A.).

### Litterfall collection and sample processing

Twelve litter traps, three on each of four transects in each plot, were deployed to estimate annual litterfall production. The four transects were parallel, each 20 to 30 m, and separated by 5 to 10 m depending on the plot size. Three litter traps 6 to 15 m apart were established on each transect. The litter traps were cylindrical and made of a nylon sheet with a mesh size of 2 mm, a collecting area of 0.283 m^2^, and a depth of 0.5 m, and were set up at 1 m height from the forest floor with three poles. The litter traps were installed in March 2011 and were emptied in July, November, and December 2011, March, May, August, and November 2012, and April 2013. The litter was separated into leaves, twigs (< 1 cm diameter), and others including bark and reproductive parts, and leaves were further sorted according to tree species. The sorted litterfall was oven-dried at 65°C for 72 h and then weighed. Annual litterfall was reported for March 2011 to March 2012 and March 2012 to April 2013.

### Fine root measurements

#### Measurement of fine root biomass

Fine root (< 5 mm) biomass was determined by soil core sampling to a depth of 30 cm with a cylindrical stainless steel corer (5 cm diameter × 60 cm length). Ten soil cores (90 in total) were excavated from randomly selected points in each plot in July 2011. The collected soil core samples were transported to the laboratory and refrigerated for several days until processing. Woody fine roots were picked out and carefully washed with tap water. Living and dead roots were separated by visual inspection based on color, texture, and shape as described by Vogt and Persson [48]. Contrary to living roots, dead roots were dark brown or black in color, had fragmented bark, and were easily broken. The roots were oven dried at 65°C for 72 h and weighed. Fine root biomass was used to calculate the fine root turnover rate.

#### Estimation of fine root production and root turnover rate

The ingrowth core method was used to estimate fine root (< 5 mm) production. In this method, fine root growth into a root-free soil in situ is measured to estimate the production [30,48]. In total, 288 ingrowth cores were installed in April 2011 by excavating 32 soil cores (5 cm diameter × 30 cm depth) at each plot. The excavated soil was separated by soil horizon and the roots were removed by sieving and hand picking. Each hole was filled with the root-free soil according to the soil horizons. The center of ingrowth cores was marked with flags for re-sampling. Eighteen ingrowth cores per visit were collected from plots using a cylindrical stainless steel corer to a depth of 30 cm. Sampling was done at one-month intervals for two years, starting from August 2011, except during the winter season (December to February) because of snow and frozen soil in the research area during this period. Fine roots in ingrowth samples were processed as described for the soil core samples.

Fine root production was calculated by using the simplified decision matrix (Table 2) of Yuan and Chen [49], modified from Fairley and Alexander [50]. We calculated the fine root turnover rate as the annual fine root production divided by the maximum standing crop of fine root biomass from soil cores [51].

**Table 2:** Simplified decision matrix for calculating production, mortality and decomposition of root [49].

*If*	*Production*	*Mortality*
*DB + DN ≥ 0 and DN ≥ 0*	*DB + DN*	*DN*
*DB ≥ 0 and DN ≤ 0*	*DB*	*0*
*DB ≤ 0 and DB + DN ≤ 0*	*0*	*|DB|*

*D* = changes in root biomass or necromass, *B* = root biomass, *N* = root necromass.

### Estimation of net primary production (NPP)

We defined NPP as the sum of woody biomass production, litterfall production, and fine root production. Woody biomass increments of stems, branches and coarse roots were estimated by the allometric models based on DBH increments of each individual (Table 1) [35]. Litterfall and fine root production were produced as described above.

### Statistical analysis

All statistical analyses were performed using SAS 9.2 software (SAS Institute Inc., Cary, NC, U.S.A.). Fine root and litterfall data were log transformed to meet normal distribution. Two-way analysis of variance (ANOVA) was performed to detect the effects of the plots and soil horizons on soil properties, followed by one-way ANOVA to test for the differences in soil properties among the plots in each soil horizon, since no interactions among the effects were detected. We used two-way ANOVA to test for the effects of year and stand on litterfall fraction and fine root production. When there is no interaction, one-way ANOVA was performed to test for significant differences in litterfall fraction and fine root production among stands in 2011 and 2012, respectively. Multiple comparisons of means were completed using Duncan’s multiple range test at α = 0.05. We used linear regression analysis to test the relationship between aboveground and belowground growth parameters.

## Results

### Soil properties

Most of the soil physical and chemical properties in the nine stands were significantly different among the stands and the soil horizons A and B (Table 3). The soil acidity and exchangeable soil Ca^2+^ and Mg^2+^ did not significantly differ among the stands and soil horizons. The soil bulk density was significantly higher in the B than in the A horizon, while chemical properties such as OM, TN, AP, and CEC were higher in the A than in the B horizon (*p* < 0.05). The differences in the soil properties among the stands were significant, but no significant difference was found between the deciduous and coniferous forests. TN and OM were highest in stands 2 and 6, whereas the lowest levels for these parameters were observed in stands 4 and 9 (*p* < 0.05). AP was significantly different among the stands (*p* < 0.01), showing the highest value in stand 6 followed by stand 2. CEC and exchangeable soil K^+^ were significantly different among stands (*p* < 0.05 and *p* < 0.01, respectively), with the highest value of K^+^ noted for stand 3 and the lowest for stand 7.

**Table 3:** Soil properties in the nine GEF study stands.

		Physical properties								Chemical properties																	
		Sand		Silt		Clay		Bulk density		pH		OM		TN		AP		CEC		Exchangeable cations							
																				K^+^		Na^+^		Ca^2+^		Mg^2+^	
		(%)		(%)		(%)		(g cm^-3^)				(%)		(%)		(mg kg^-1^)		(cmolc kg^-1^)		(cmolc kg^-1^)							
A horizon																											
Deciduous	1	33.4	^b^	44.2	^a^	22.3	^ab^	0.93	^abc^	4.85	^a^	4.72	^ab^	0.19	^ab^	4.83	^b^	14.74	^ab^	0.20	^abc^	0.04	^a^	0.87	^a^	0.28	^a^
	2	43.3	^ab^	38.2	^ab^	18.5	^abc^	0.82	^bc^	4.77	^a^	8.85	^a^	0.38	^a^	14.45	^ab^	20.45	^a^	0.27	^abc^	0.03	^a^	2.11	^a^	0.50	^a^
	3	51.8	^ab^	32.0	^ab^	16.2	^abc^	1.19	^ab^	5.17	^a^	3.44	^ab^	0.14	^b^	4.31	^b^	10.67	^b^	0.33	^a^	0.04	^a^	1.04	^a^	0.24	^a^
	4	46.3	^ab^	31.9	^ab^	21.8	^ab^	1.17	^ab^	4.85	^a^	3.25	^ab^	0.08	^b^	2.34	^b^	10.12	^b^	0.26	^abc^	0.03	^a^	0.16	^a^	0.17	^a^
	5	37.9	^b^	42.1	^a^	19.9	^abc^	1.01	^abc^	4.94	^a^	4.35	^ab^	0.15	^b^	5.19	^b^	11.53	^b^	0.18	^bc^	0.03	^a^	0.24	^a^	0.12	^a^
Coniferous	6	50.1	^ab^	35.4	^ab^	14.5	^bc^	0.94	^abc^	5.10	^a^	8.38	^a^	0.26	^ab^	26.64	^a^	15.18	^ab^	0.22	^abc^	0.04	^a^	2.93	^a^	0.31	^a^
	7	40.9	^b^	35.6	^ab^	23.5	^a^	0.71	^c^	5.03	^a^	5.22	^ab^	0.16	^b^	2.42	^b^	13.59	^ab^	0.15	^c^	0.03	^a^	0.14	^a^	0.10	^a^
	8	62.6	^a^	24.7	^b^	12.7	^c^	1.28	^a^	5.22	^a^	3.56	^ab^	0.10	^b^	5.84	^b^	9.63	^b^	0.30	^ab^	0.03	^a^	2.52	^a^	0.36	^a^
	9	48.2	^ab^	31.4	^ab^	20.4	^abc^	1.23	^ab^	5.06	^a^	2.26	^b^	0.06	^b^	3.25	^b^	10.21	^b^	0.23	^abc^	0.03	^a^	0.35	^a^	0.10	^a^
B horizon																											
Deciduous	1	38.5	^b^	35.8	^a^	25.7	^ab^	0.96	^abc^	4.94	^a^	2.83	^ab^	0.11	^ab^	0.56	^b^	12.71	^ab^	0.14	^abc^	0.06	^a^	0.68	^a^	0.32	^a^
	2	46.0	^ab^	36.8	^ab^	17.2	^abc^	0.89	^bc^	4.90	^a^	5.99	^a^	0.23	^a^	6.33	^ab^	14.17	^a^	0.18	^abc^	0.04	^a^	1.82	^a^	0.35	^a^
	3	52.9	^ab^	28.6	^ab^	18.5	^abc^	1.20	^ab^	5.14	^a^	1.55	^ab^	0.05	^b^	1.84	^b^	9.11	^b^	0.43	^a^	0.04	^a^	0.85	^a^	0.31	^a^
	4	49.0	^ab^	28.7	^ab^	22.3	^ab^	1.32	^ab^	4.95	^a^	0.98	^ab^	0.03	^b^	1.55	^b^	9.56	^b^	0.19	^abc^	0.04	^a^	0.17	^a^	0.41	^a^
	5	47.5	^b^	36.0	^a^	16.5	^abc^	1.40	^abc^	5.21	^a^	1.37	^ab^	0.04	^b^	1.27	^b^	8.56	^b^	0.14	^bc^	0.04	^a^	0.40	^a^	0.20	^a^
Coniferous	6	62.5	^ab^	24.8	^ab^	12.7	^bc^	1.10	^abc^	5.18	^a^	5.52	^a^	0.15	^ab^	10.42	^a^	10.51	^ab^	0.15	^abc^	0.03	^a^	1.19	^a^	0.48	^a^
	7	46.2	^b^	32.5	^ab^	21.4	^a^	1.11	^b^	5.28	^a^	2.71	^ab^	0.07	^b^	1.80	^b^	10.44	^ab^	0.10	^c^	0.03	^a^	0.16	^a^	0.13	^a^
	8	58.2	^a^	25.7	^b^	16.1	^b^	1.30	^a^	5.56	^a^	2.91	^ab^	0.05	^b^	2.44	^b^	8.99	^b^	0.32	^ab^	0.04	^a^	2.28	^a^	0.32	^a^
	9	52.6	^ab^	24.9	^ab^	22.5	^abc^	1.23	^ab^	5.09	^a^	1.26	^b^	0.04	^b^	1.75	^b^	10.00	^b^	0.33	^abc^	0.03	^a^	0.79	^a^	0.26	^a^

OM = organic matter; TN = total nitrogen; AP = available phosphorus (P_2_O_5_); CEC = cation exchange capacity. Mean values with the same superscript letters are not significantly different among the nine stands within each soil horizon (*p* < 0.05; Duncan’s multiple range test).

### Litterfall

Annual total litterfall mass was significantly different across the stands (*p* < 0.01), but not between years (*p* = 0.17), ranging from 360 to 651 g m^-2^ yr^-1^ in 2011 and from 300 to 656 g m^-2^ yr^-1^ in 2012 (Table 4). Leaf litter formed the major fraction of litterfall in all stands, representing 74% on average of the total litterfall mass per stand in 2011 and 62% in 2012. Twigs up to 1 cm in diameter accounted for 9% and nonleaf including bark and reproductive parts were 17% of total litterfall in 2011, and 18 and 20% in 2012.

**Table 4:** Annual litterfall mass (g m^-2^) sorted into three fractions (leaves, twigs, and others).

Year	Forest type	Stand	Leaves			Other leaves			Twigs			Nonleaf			Total		
2011	Deciduous	1	331	(19)	^abcd^				56	(11)	^ab^	78	(9)	^b^	465	(27)	^c^
		2	264	(37)	^de^				25	(7)	^c^	71	(4)	^b^	360	(40)	^d^
		3	349	(10)	^abc^				69	(11)	^a^	85	(9)	^b^	503	(27)	^bc^
		4	311	(9)	^bcd^				42	(4)	^abc^	61	(3)	^b^	415	(9)	^cd^
		5	301	(7)	^cd^				40	(11)	^bc^	94	(12)	^b^	435	(20)	^cd^
	Coniferous	6	363	(22)	^abc^	5	(3)	^c^	68	(5)	^a^	59	(7)	^b^	495	(29)	^bc^
		7	407	(29)	^a^	98	(24)	^b^	46	(8)	^abc^	43	(12)	^b^	594	(33)	^ab^
		8	204	(37)	^e^	182	(36)	^a^	28	(8)	^c^	70	(15)	^b^	484	(45)	^c^
		9	387	(13)	^ab^	37	(6)	^bc^	28	(4)	^c^	199	(36)	^a^	651	(33)	^a^
2012	Deciduous	1	308	(12)	^cd^				100	(23)	^a^	107	(11)	^bc^	516	(28)	^bc^
		2	279	(9)	^de^				99	(13)	^a^	74	(17)	^cd^	452	(20)	^c^
		3	363	(16)	^b^				115	(17)	^a^	178	(15)	^a^	656	(42)	^a^
		4	418	(6)	^a^				117	(13)	^a^	91	(10)	^bcd^	625	(24)	^ab^
		5	322	(6)	^bcd^				93	(8)	^a^	203	(37)	^a^	618	(39)	^ab^
	Coniferous	6	254	(25)	^e^	9	(3)	^c^	112	(15)	^a^	70	(1)	^cd^	445	(40)	^c^
		7	87	(19)	^f^	112	(20)	^b^	57	(29)	^a^	44	(13)	^d^	300	(58)	^d^
		8	107	(16)	^f^	189	(36)	^a^	67	(25)	^a^	58	(7)	^cd^	420	(38)	^c^
		9	345	(10)	^bc^	49	(2)	^bc^	51	(6)	^a^	127	(12)	^b^	571	(21)	^ab^

Leaf litter of species other than the planted tree species in coniferous forests were not separated and are called “Other leaves”. Nonleaf includes bark, reproductive parts and unidentified organic matters. Parentheses represent standard error (n = 4). Mean values with the same superscript letters are not significantly different within each year among the nine stands.

**Fig 2:**
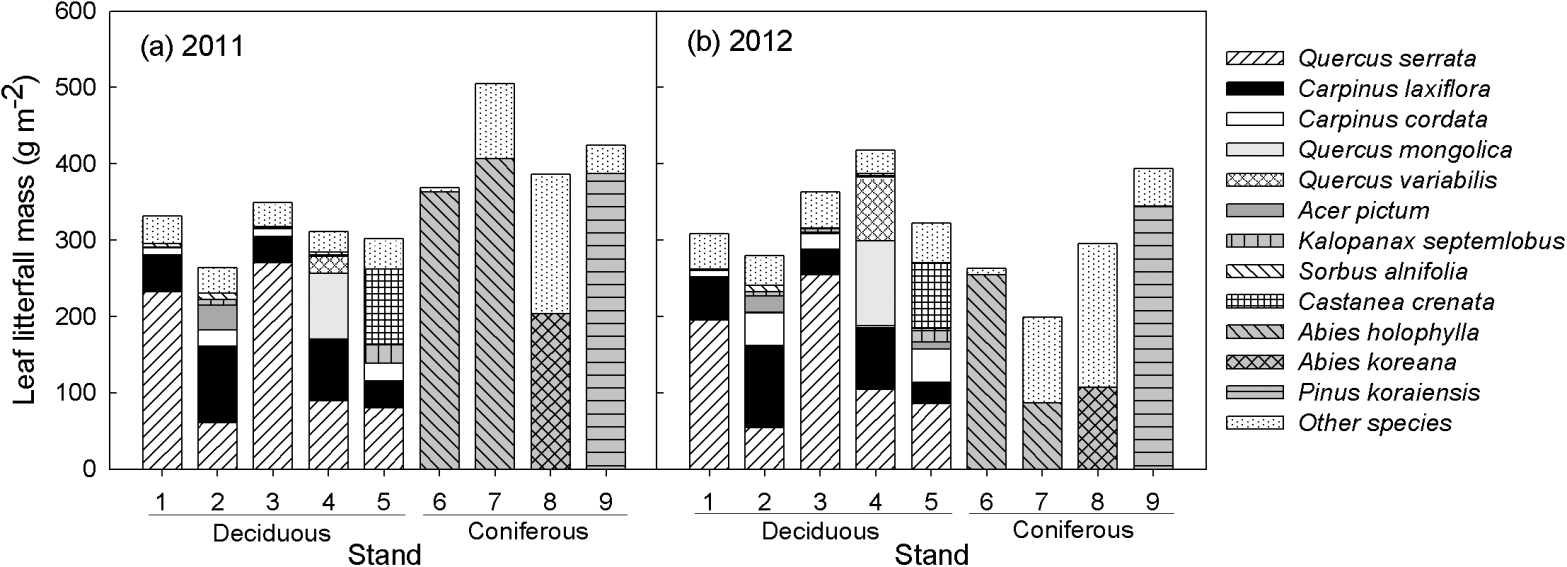
Annual leaf litter mass in 2011 and 2012, stacked by species, for each of the nine GEF stands. Other species indicates leaf litter from tree species other than the nine most prevalent species in deciduous forests and naturally regenerated species not planted in coniferous forests.

In deciduous forests (stands 1–5), the nine most prevalent tree species (*Q*. *serrata*, *C*. *laxiflora*, *C*. *cordata*, *Q*. *mongolica*, *Q*. *variabilis*, *A*. *pictum*, *K*. *septemlobus*, *S*. *alnifolia*, and *C*. *crenata*) accounted for 88% on average of the leaf litterfall mass. The sum of leaf litter from *Q*. *serrata* and *C*. *laxiflora* accounted for the highest proportion of total leaf litter with an average of 63% (Fig 2). In coniferous forests (stands 6–9), leaf litterfall from naturally regenerated deciduous trees accounted for 1.4–47.1% in 2011 and 3.5–67.5% in 2012.

### Fine root mass, production and turnover rate

**Fig 3:**
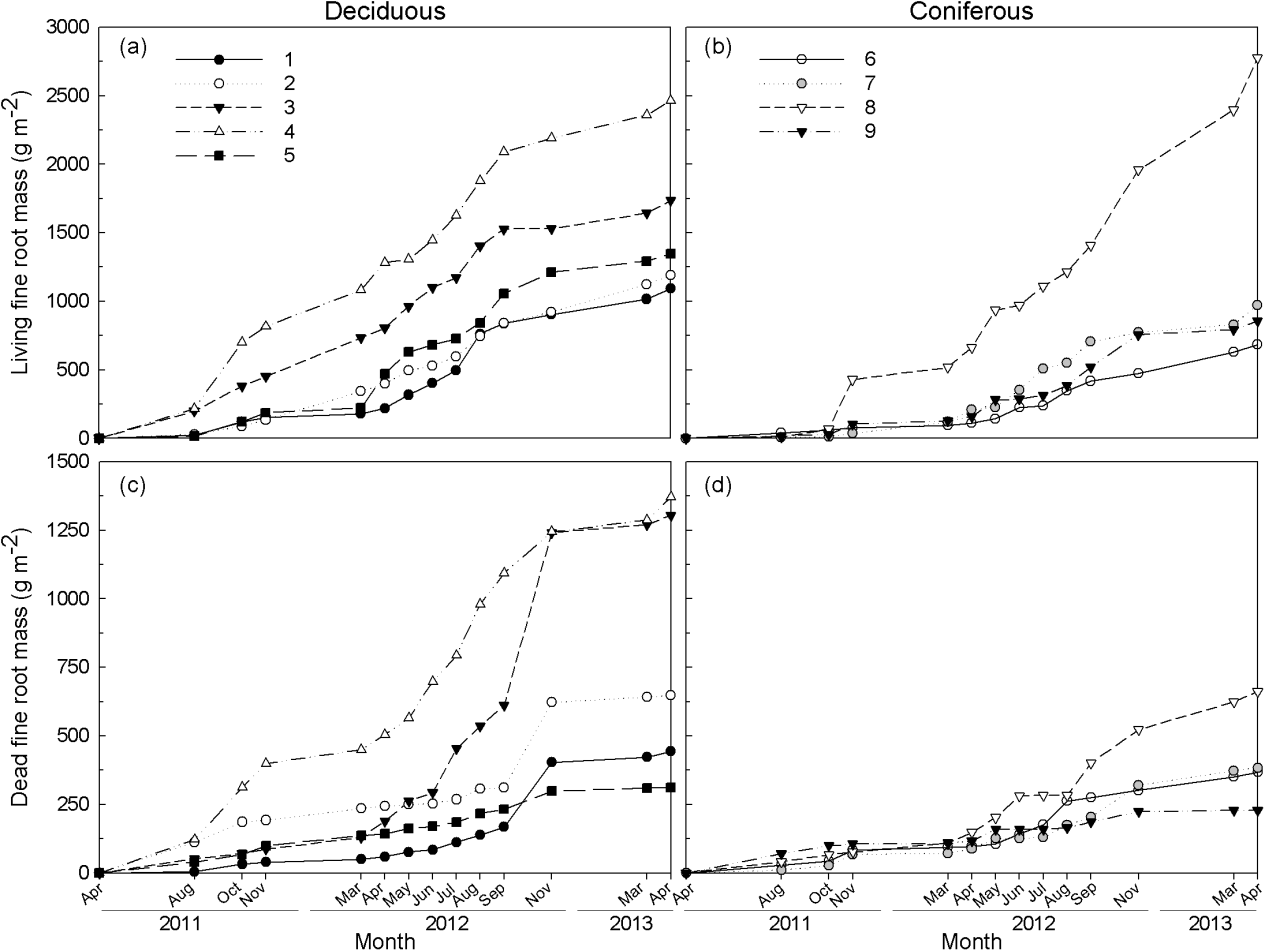
Living ((a) and (b)) and dead ((c) and (d)) fine root mass in ingrowth cores during two years from April 2011 to April 2013 in five deciduous ((a) and (c)) and four coniferous ((b) and (d)) forests at the GEF, Korea.

**Fig 4:**
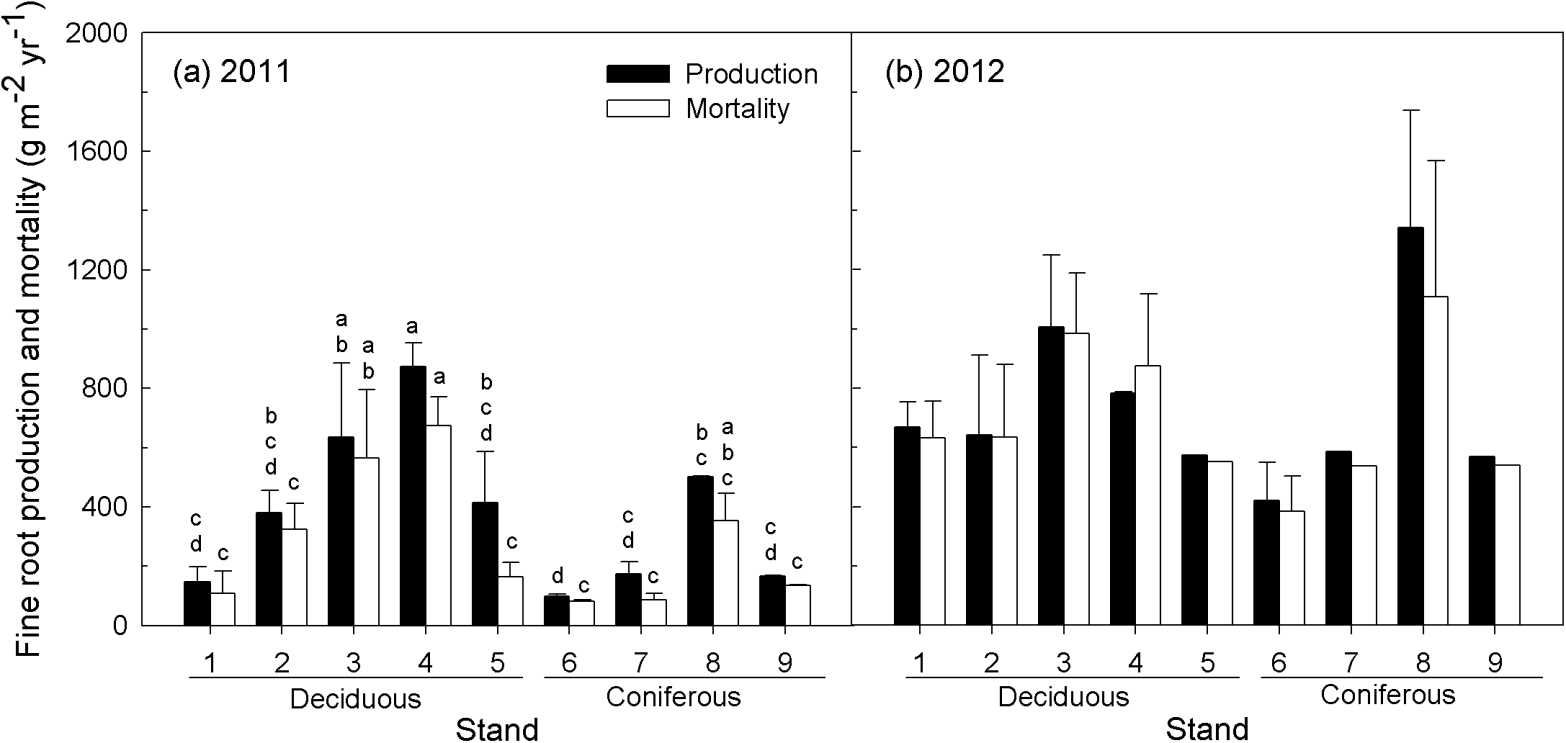
Annual fine root production and mortality in the nine stands in (a) 2011 and (b) 2012, estimated with the ingrowth core method. Vertical bars represent standard errors. Different letters above the vertical bars denote significant differences between the means of different stands (α = 0.05; Duncan’s multiple range test).

Fine root biomass during the first two years ranged from 1092 to 2465 g m^-2^ in deciduous forests and from 685 to 2774 g m^-2^ in coniferous forests (Fig 3). Annual fine root production and mortality estimated by the ingrowth core method were higher in 2012 than in 2011 (Fig 4). Annual fine root production ranged from 99 to 872 g m^-2^ yr^-1^ with significant differences among the stands (*p* < 0.01) in 2011. Annual fine root mortality ranged from 82 to 674 g m^-2^ yr^-1^ with significant differences among stands in 2011 (*p* = 0.05). Fine root production and mortality were not significantly different among the stands in 2012.

Fine root turnover (Table 5) was significantly higher in 2012 than in 2011 (*p* < 0.01). The differences among stands were large, almost an order of magnitude, but there were no statistically significant differences because the variability was so high.

**Table 5:** Fine root turnover rates in 2011 and 2012 across the nine stands.

Forest type	Stand	Fine root turnover rate (yr^-1^)	
		2011	2012
Deciduous	1	0.30	1.35
	2	0.87	1.48
	3	1.94	3.06
	4	1.91	1.72
	5	1.63	2.25
Coniferous	6	0.42	1.79
	7	0.38	1.29
	8	2.05	5.49
	9	0.97	3.34

### Above- and belowground contribution to NPP

Our results showed that fine roots constituted 18–44% of the annual NPP and the fraction was higher in deciduous forests than in coniferous forests, but was not significantly different (*p* = 0.29). The sum of litterfall and fine root production contributed 62% of the NPP in deciduous and 55% in coniferous forests in this study.

**Fig 5:**
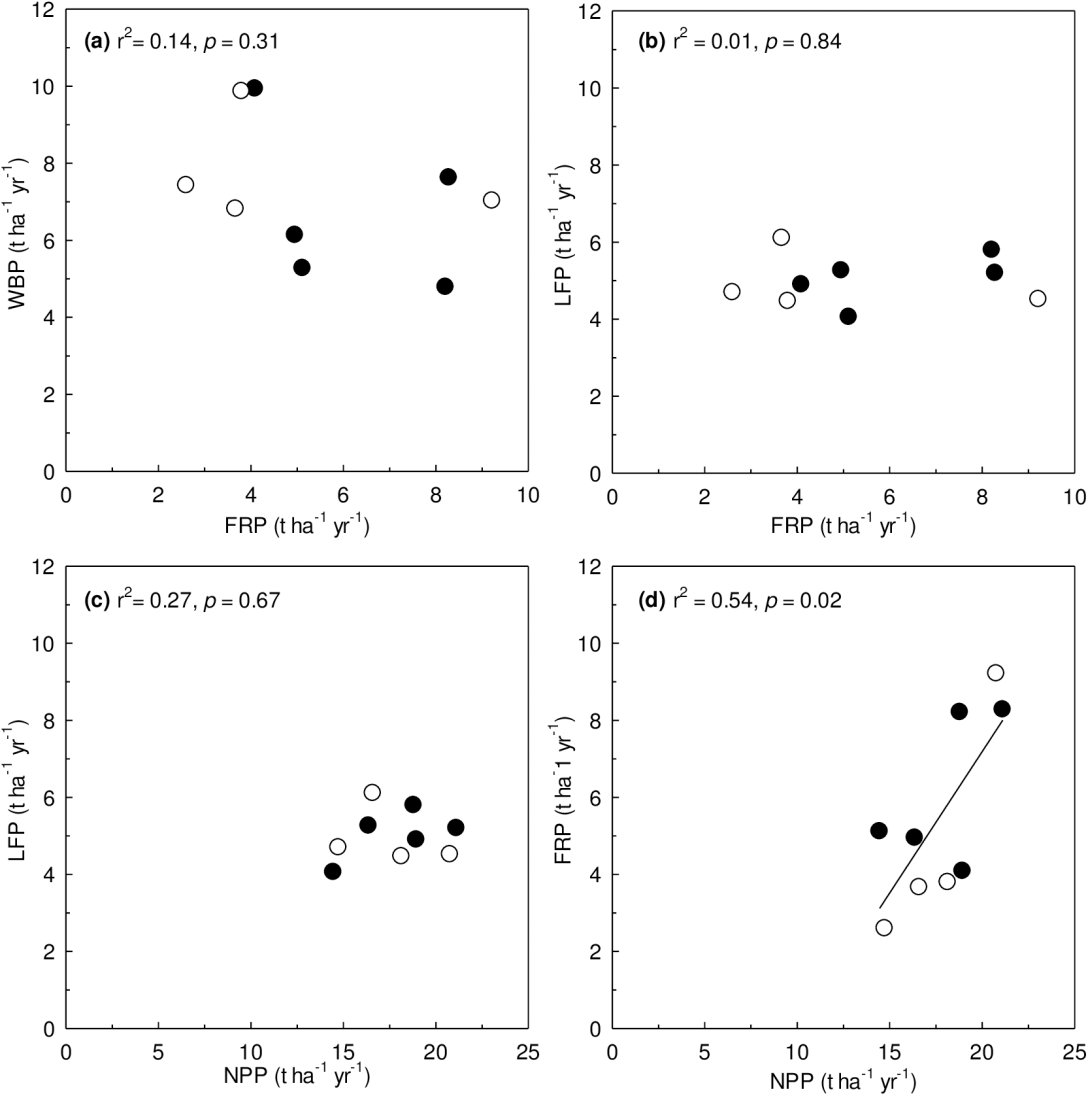
Relationships between the components of NPP across deciduous and coniferous forests in GEF. WBP = woody biomass production; LFP = litterfall production; NPP = net primary production; FRP = fine root production. Symbols are: ● = deciduous forests in GEF, ○ = coniferous forests in GEF.

There was no significant relationship of woody biomass production (r^2^ = 0.14, *p* = 0.31) or litterfall mass (r^2^ = 0.01, *p* = 0.64) to fine root production. Litterfall mass was not significantly correlated with NPP (r^2^ = 0.27, *p* = 0.67), but fine root production was (r^2^ = 0.54, *p* = 0.02; Fig 5). In other words, a greater fraction of carbon was allocated to root production in more productive forests.

## Discussion

### Fine root production and root turnover rate

The ingrowth core method is a useful tool for among-site comparison of fine root growth [52,53]. The simplified decision matrix used in this study provided mathematical logic for the development of the matrix and provided simplified equations to facilitate computation for large datasets of root production and mortality [49]. Annual fine root production in this study was within the same range as previously reported for temperate forests, regardless of the measurement and calculation methods used [24,30,32,54]. Tateno et al. [19] similarly reported a fine root production of 90–680 g m^-2^ yr^-1^, with a mean of 360 g m^-2^ yr^-1^, which was estimated by the ingrowth core method in cool-temperate broad-leaved deciduous forests. Van do et al. [55] reported relatively low fine root production of 135 g m^-2^ yr^-1^ in a *Q*. *serrata* pure plantation and it was comparable to fine root production of stand 1 in 2011 which is the lowest estimate in deciduous stands.

In the coniferous forests (stands 6–9), the annual fine root production ranged from 99 to 501 g m^-2^ yr^-1^ in 2011, and 421 to 1342 g m^-2^ yr^-1^ in 2012, with the maximum fine root production noted for stand 8. Noguchi et al. [56] reported annual fine root production of 72–320 g m^-2^ yr^-1^ for *Cryptomeria japonica*, 122–306 g m^-2^ yr^-1^ for *Chamaecyparis obtusa*, 7–13 g m^-2^ yr^-1^ for *Pinus thunbergii*, and 90–378 g m^-2^ yr^-1^ for temperate deciduous broadleaved forests in Japan. Fine root production of coniferous trees in 2011 was similar with that of Japanese larch reported in previous Korean studies (111–210 g m^-2^ yr^-1^; [57,58]). The high fine root production observed in stand 8 might be induced by the understory vegetation as the leaf litter mass of the understory vegetation in this stand accounted for 49% of total leaf litterfall mass. A high proportion of the fine roots of understory vegetation among total fine roots in coniferous plantation has previously been reported [59]. Lower production has commonly been observed in the first year after installation of ingrowth cores than in the following years [60–62]. One of the reasons for underestimation in the first year of ingrowth core may be retarded root re-colonization. Vogt and Persson [48] stated that root re-colonization may not start until 6–9 months after installation of ingrowth cores. Makkonen and Helmisaari [52] also reported that the fine root production in ingrowth cores at three years after installation was similar to that estimated with sequential soil cores in Scots pine stands in eastern Finland. We, too, found that fine root production was lower in 2011 than in 2012 resulting in a lower turnover rate in that year.

The rates of fine root turnover in this study were generally comparable to those reported by Gill and Jackson [51] except for a few outliers that showed large values in conifer stands of *A*. *koreana* and *P*. *koraiensis* in 2012 due to relatively high fine root production. They performed a meta-analysis of 190 published studies to analyze global patterns in root turnover in terrestrial ecosystems and reported a fine root turnover rate ranging at 0.02–2.64 yr^-1^ with an average of 0.52 yr^-1^. In addition, a turnover rate of 0.1–2.0 yr^-1^ has been reported for deciduous forests and of 0.5–0.7 yr^-1^ for coniferous forests in temperate ecosystems [24,63,64].

### Litterfall and fine root contribution to NPP

Litterfall and fine roots are major carbon fluxes (34% for litterfall and 25% for fine roots of NPP, on average) across deciduous and coniferous forests in this study. In a warm-temperate evergreen broadleaved forest of Japan, Do et al. [1] reported 39% litterfall and 33% fine root production of NPP. Nadelhoffer et al. [24] reported that the fraction of NPP allocated to leaf litter and fine roots together was 53% on average at nine temperate forest sites. In other studies, the ratio of fine root production to annual NPP ranged from 8 to 76% [15,30,65,66].

There were no significant relationships between above- and belowground production across the nine stands in this study. Consistent with this result, no overall pattern was observed between aboveground production and fine root production in a 43 forest sites worldwide [30]. In contrast, Hendricks et al. [21] reported that fine root production estimated by using a minirhizotron was positively related with foliage production estimates across soil resource availability gradients in nine temperate forests.

We found that belowground production was a greater fraction of NPP where NPP was high, which indicates greater carbon allocation belowground in more productive forests (Fig 5). However, Tateno et al. [19] reported that the ratio of belowground production to NPP decreased with increasing soil N availability. Nadelhoffer [67] reviewed the uncertainties how fine root production and turnover vary in relation to soil N availability and suggested four possible relationships based on declining fine root biomass along N availability. The last one was that the proportional allocation of total primary production to fine roots decreases as fine root turnover and production increase with N availability. However, the proportion of NPP allocated to fine roots could increase if fine root turnover rates and production increase as he hypothesized and fine root biomass is concurrently unrelated with N availability as observed in some forests [68–70]. We found a relationship with NPP, which is influenced by other environmental factors in addition to nutrient availability, such as water availability, topographic position, and species composition. Because our result is based on only 9 stands in Korea, there should be further studies in various types of forests to generalize the relationship of carbon allocation between above- and belowground across NPP gradients in forest ecosystems.

## Conclusions

Litterfall, fine root production, and fine root turnover rate differed across the nine stands and varied between the two sampling years as well. We confirmed that the magnitude of carbon flux in fine root turnover in these temperate forests is similar to that of litterfall. Although we could not find a significant relationship between above- and belowground production, our results contribute to the understanding of carbon allocation between above- and belowground in temperate forests: belowground production was a greater fraction of NPP where NPP was high, which indicates a greater carbon allocation belowground in more productive forests. If this finding proves to be generally true, it could be used to improve the accuracy of simulation models for carbon cycling in forest ecosystems.
